# Data from CREP Replication of Does Money Buy Happiness, Diener et al. (2010)

**DOI:** 10.5334/jopd.156

**Published:** 2026-06-15

**Authors:** Olivia Costa, Quincey Feragen, Jordan Wagge, Amanda M. Woodward

**Affiliations:** 1 University of Minnesota Twin Cities, United States; 2 Avila University, United States

**Keywords:** Happiness, Well-Being, Basic Needs, Psychological Needs, Replication

## Abstract

This dataset results from a multi-lab replication of Diener et al. (2010) organized by the Collaborative Replication and Education Project (CREP). This project aimed to understand the relationships between different types of needs (basic needs, psychological needs, standard of living, etc.), people’s positive and negative feelings, and life evaluations. This dataset includes data collected across each site contributing to the study. It includes predictor variables associated with participants’ mood and current financial comfort. It also includes demographic data like education, gender, marital status, residential area and income. The data are stored in a. csv file to be used for additional analysis, as an educational tool, and further interdisciplinary collaboration.

## (1) Background

Research into the relationship between money and happiness has yielded mixed results (Killingsworth et al., 2022, p. 1; Ford et al., 2016). One reason for these discrepancies may be differences in study designs, such as sample characteristics and how income and well-being are measured. In fact, at least one replication study has demonstrated that the relationship between money and happiness depends on methodology used in the study and, when the relationship exists, it may be weak (Aknin et al., 2020). Further, many studies have focused solely on the relationship between income and positive affect, though some researchers suggest that a more complete answer may lie in analyzing negative affect as well (Killingsworth et al., 2022).

Diener and colleagues (2010) aimed to provide a broader understanding of the relationship between income and well-being by incorporating multiple indicators of happiness. Rather than relying on a single measure, the study included life evaluations, positive and negative emotions, income, and material possessions. This multi-faceted approach allows for a more nuanced exploration of how money and happiness may be connected (Disabato et al., 2016, p. 475). Prior research has emphasized that the relationship between income and psychological well-being is not unidirectional and may be influenced by unmet psychological needs and access to mental health resources (Srivastava et al., 2001, p. 961; O’Donnell et al., 2021). Diener and colleagues (2010) included measures of both emotional states and life satisfaction, offering a unique opportunity to further explore these bidirectional dynamics.

The data in this dataset come from a multi-lab collaboration supported by the Collaborative Replication and Education Project (Project CREP; Wagge, 2019) designed to replicate the original Diener et al (2010) study. This dataset is a combination of data from several sites and were collected primarily by teams of undergraduate researchers. The dataset includes variables related to basic and psychological needs and individual wellbeing. Additionally, these data include demographic information collected from participants at different study sites and income adjusted to United States Dollar values from October 2023 to account for any differences in currency across multiple currencies and multiple time points.

Data were collected by the following Institutions all over the world: University of Vienna – Austria, Illinois Institute of Technology in Chicago Illinois, Marian University of Indianapolis, University of Toronto, Education University of Hong Kong, Tilburg University – Netherlands, and Pacific Lutheran University (as seen in Table 2). Materials were provided to research teams via an OSF page (https://osf.io/px0sw/). Participating research teams forked OSF project pages and stored all materials and data on these pages. Following CREP procedures, projects were reviewed prior to data collection. Some projects included additional measures (a “direct+” replication); teams describe these additional measures on their project wikis. Any additional measures were required to be added at the end of the survey.

The data presented in this paper can be used for both research and educational purposes. Researchers may find this dataset helpful to conduct exploratory analyses on predictors of life satisfaction and educators may find this dataset useful for in-class examples in statistics or methods courses. For instance, the data could be used in statistics courses when learning about correlation and regression. That way, students can form hypotheses about the relationship between happiness and wealth and then test the relationship with the data. Educators may also use these data in social psychology or wellbeing courses, as they can be used to demonstrate how researchers study these topics.

## (2) Methods

### 2.1 Study Design

This dataset was created by combining data from all replications of Diener et al (2010) through Project CREP. Overall, 58 different research teams created forks of the Open Science Framework Page (https://osf.io/px0sw/). Twenty-one of these replications were publicly accessible. To ensure we had as much data as possible, we reached out to research groups without publicly accessible data, though most of these contact attempts were unsuccessful. Of the 21 publicly available replications, 11 were excluded for the following reasons: ten did not provide any data or summary statistics and one was missing a codebook, which made the data uninterpretable. Descriptions of the included replication study sites are available in Table 1. One study site collected data via phone calls, and the remaining sites collected data online.

**Table 1:** Replication Study Sites and sample sizes.


STUDY SITES	DATE COLLECTED	SURVEY MODALITY	*N*

(1) Replication of Diener et al. (2010) at University of Toronto, tut5401, W19 (Tian et al., 2024)	Apr. 2019	Online	57

(2) Wealth and Happiness: Replication of Diener, E., Ng, W., Harter, J., & Arora, R. (2010) at University of Vienna (Müller et al., 2021)	Oct. 2020	Online	163

(3) Replication of Diener, Ng, Harter, & Arora (2010) at IIT Spring 2018 (Pys, P. M et al., 2021)	Jan. 2018	Online	213

(4) Wealth and Happiness: Replication of Diener, et al. at IIT 2019 (Legate et al., 2022)	Mar. 2019	Online	183

(5) Replication of Diener et al. (2010) at Marian University (Reed et al., 2020)	Jan. 2020	Online	127

(6) Replication of Diener et al. (2010) at Education University of Hong Kong (Wong et al., 2019)	Dec. 2019	Phone	200

(7) (Pierce County) Replication of Diener E., Ng W., Harter J., & Arora R. (2010). for Collaborative Replications and Education Project (Hatton et al., 2019)	Jan. 2014	Phone	100

(8) CREP Diener Replication Project. Tutorial Section TUT5401. W19 (Merkley et al., 2019)	Apr. 2019	Online	29

(9) Replication of Diener, et al. (2010). at Pacific Lutheran University (Grahe et al., 2020)	Apr. 2019	Online	99

Total N			1171


*Note*. The table includes information about all replication sites, survey modality and sample size. An OSF link is included for each specific page.

All data collection sites followed a similar procedure. Sites around the world volunteered to directly replicate Diener et al (2010) using the same materials available on the CREP OSF page (https://osf.io/px0sw/overview). Specifically, all participants completed an income measure, and surveys related to their basic and psychological needs. Some sites chose to include extension hypotheses and included additional measures to test these hypotheses following the original study. Prior to data collection, the site’s procedure was reviewed by two CREP reviewers and an executive team member to ensure that the direct replication was occurring consistently across sites.

### 2.2 Time of data collection

The replications occurred between 2014 and 2018 (see Table 1). We aggregated the data across sites in October 2023.

### 2.3 Location of data collection

The replications included in this dataset were collected from several different Universities throughout the United States, Canada, Austria, Netherlands, and China. The four replications in the United States were from Marian University in Indianapolis, Indiana, Pacific Lutheran University in Tacoma, Washington, and two of the four replications were from the Illinois Institute of Technology in Chicago, Illinois. There were two replications from the University of Toronto, Canada. The rest of the replications were from the Education University of Hong Kong, China, the University of Vienna, Austria, and Tilburg University, The Netherlands. The aggregated dataset was combined at the University of Minnesota – Twin Cities in October 2023.

### 2.4 Sampling, sample, and data collection

The overall sample contained 1,171 participants. Demographic information for the sample is reported in Table 2. While most study sites reported demographic information, several study sites opted out of reporting some demographic data. Study sites 4 and 6 did not report age in their datasets. Study sites 4, 5, and 6 did not report the marital status or residential area of their participants and study sites 6 and 7 did not report education attained. Lastly, study site 9 did not report marital status using the key from the Diener et al. (2010) paper, thus it has been omitted from the martial status table. We could not contact the authors, and marital status was not included in their analyses on OSF.

**Table 2:** Demographics of total study sites.


CHARACTERISTICS	CATEGORY	FREQUENCY	PERCENTAGE

Gender	Man	617	52.7

	Woman	512	43.7

	Nonbinary	4	0.3

	No response	38	3.2

Marital Status	Divorced/Separated	2	0.17

	Domestic Partnership	144	12.3

	Married	23	1.9

	Single	431	36.8

	NA	472	40.3

Residential Area	Rural	34	2.9

	Suburban	172	14.6

	Urban/City	485	41.4

	Village	20	1.7

	NA	460	39.3

Education	Graduate	41	3.5

	Full Tertiary	208	17.8

	Some Tertiary	461	39.4

	Secondary	163	13.9

	Primary or less	31	2.6

	NA	259	22.1

	Other	8	0.68


*Note*. The table includes information about all replication sites, gender, marital status, residential area, and education.

Subsequently, 958 participants reported income, 81.8% of the total sample. The proportion of participants reporting income varies across study sites (see Table 3). Median annual income (USD), calculated among those reporting income, ranged from $3,650 ($0 – $43,800) to $80,000 ($40,000 to $125,000). Currency across study sites were adjusted to the equivalent United States dollar value in October 2023.

**Table 3:** Descriptive statistics for total income and income per study site.


STUDY SITE	*N*	INCOME REPORTED *n*(%)	MEDIAN INCOME (USD)	IQR

Total	1171	958 (81.8%)	40,000	13,000–85,750

Site 1	57	57 (100%)	3,650	0–43,800

Site 2	163	139 (85.3%)	16,350	10,900–38,150

Site 3	213	150 (70.4%)	70,000	33,000–108,750

Site 4	183	137 (74.9%)	75,000	45,000–160,000

Site 5	127	79 (62.2%)	50,000	19,150–100,000

Site 6	200	193 (96.5%)	23,400	0–52,000

Site 7	100	97 (97%)	36,000	65–60,000

Site 8	29	29 (100%)	17,520	6,570–138,700

Site 9	99	77 (77.8%)	80,000	40,000–125,000


*Note*. Table 3 includes income data from the combined study sites and each individual site. The total *N* is reported for each site, as well as the total income reported *n* (%), median income (USD) and interquartile range (IQR).

### 2.5 Materials/survey instruments

The replications we included in the aggregated dataset used the same materials and surveys as the original Diener and colleagues (2010) study. More details about the surveys are available on our OSF page (https://osf.io/qdx7p).

#### Cantril Scale

The Cantril Scale was used to examine an individual’s perception of their well-being (Cantril, 1965). This measure quantifies life satisfaction by having participants imagine a ladder with 10 rungs. The top rung (10) represents the best possible life quality, and the bottom rung (1) represents the worst possible life quality. Participants are instructed to pick which rung of the ladder they feel best indicates their current level of life satisfaction. The Cantril Scale has strong convergent validity with other well-being measures across diverse samples.

#### Day Construction Method

The Day Construction Method (DCM), which evaluates how often a person spends their time doing different activities on a day-to-day basis, was used to examine how people experience their lives daily (Daniel et al., 2004, p. 1776) and is generally considered a valid survey tool in assessing well-being and daily experiences. The first DCM Survey given to participants asked six emotion related questions. Participants were instructed to respond “yes” or “no” if they remembered engaging in one of the emotions or behaviors during the previous day. For instance, participants were asked questions like, “Did you *smile* or *laugh* a lot yesterday?” and “Did you experience *depression* during a lot of the day yesterday?”.

Participants were again asked a different set of questions following the Day Construction Method format. These were five questions relating to activities and relationships that the participant engaged in the previous day. For example, “Did you learn something new yesterday?” and “Do you have family or friends you can count on in an emergency?” Both surveys aim to seek the participants’ emotional well-being, and support system.

#### Household Income Survey

This study also included questions related to household economic status. Participants were asked a single question about their households’ income and the number of people in the household. Participants also responded to three questions about their ability to access television, computer, and internet (yes = 1, no = 0). These responses were averaged together to create a single score reflecting their economic status. Participants answered two questions about access to basic needs, specifically whether they had access to food or shelter. These responses were averaged (yes = 1, no = 0). Finally, participants were asked if they were satisfied with their standard of living.

#### Assessing Short Term Financial Confidence:

In addition to the well-being measures from Diener et al. (2010), the replication protocol included two social status items (e.g., the ability to pay a $300 speeding ticket or $5000 lawsuit within a week). These questions, created as part of a replication and extension project conducted by CREP (see https://osf.io/ar5fc/), were added to the end of the questionnaire to gauge the financial stability of the participants and were calculated into their overall financial well-being.

### 2.6 Quality Control

We began reviewing various OSF forks linked to the project OSF page (https://osf.io/px0sw/). Many of these forks were inaccessible or set to private. Out of the 21 publicly available replications, we excluded 12 due to the absence of raw data, unresponsive pages, missing summary, descriptive information, or results. One additional dataset was excluded because the raw data were not properly labeled, making it impossible to determine key variables. Data cleaning was conducted using excel and RStudio, the cleaned. csv file is available on our OSF page along with the corresponding R Markdown file used for preparing the data for analysis.

### 2.7 Data anonymization and ethical issues

All data were accessed through OSF and open for public use with no identifying information in which all individual sites received IRB approval for their methods. Ethics approval or a waiver of required ethics approval was available for each site via their specific OSF page listed in Table 1.

### 2.8 Existing use of data

The dataset was used to conduct a pooled analysis that was presented as a poster at CREPCON 2024.^1^ To our knowledge, these data have not been published in any other way.

## (3) Dataset description and access

### 3.1 Repository Location

OSF General Page: https://osf.io/qdx7p/overview.

Github repository: https://github.com/odcosta20/Pooled-Data-Diener-et-al-2010.git.

### 3.2 Object/File Name

Diener_et_al_replication_data.csv.

### 3.3 Data type

The data is secondary data compiled in OSF. The data contains predominantly categorical variables with income being a numerical variable. The data contains psychological, self-report, and socio-economic measures that are all self-reported.

### 3.4 Format names and versions

The version of R used is, R version 4.5.2 (2025-10-31). Materials for this dataset are found in the OSF link above. Specifically, the Data folder inside the Methods and Materials folder. Within the Data folder, there is a PDF of the Codebook, an HTML file called Diener et al replication data cleaning, an .csv file called Diener_et_al_replication_data, and a README.rmd as well. We have also inclueded a “cleaned_diner_et_al_dataset.csv”, this is the dataset that has been cleaned after it has ran through the Rscript. Packages used are readxl, dplyr, tidyverse, ltm, ggplot2, tidyr, gridExtra, knitr, markdown, and kableExtra.

### 3.5 Language

English

### 3.6 License

CC-By Attribution 4.0 International

### 3.7 Limits to sharing

N/A

### 3.8 Publication date

The data was made public on OSF on 12 November 2023.

### 3.9 FAIR data/Codebook

To adhere to the FAIR guidelines—Findable, Accessible, Interoperable, and Reusable— when utilizing this data, we have undertaken the following actions.

#### Findability

To ensure the data and necessary materials are findable, a public repository has been created in Open Science Framework for this project titled *Compilation of Diener Et Al*. *(**2010**) OSF Multi-Site Replication Projects*. It can be accessed directly at https://osf.io/qdx7p/ as well as assigned a DOI: 10.17605/OSF.IO/QDX7P. The repository includes appropriate metadata, keywords, and subject categories to aid with indexing and visibility through repository search capability. Additionally, there is a Github repository (https://github.com/odcosta20/Pooled-Data-Diener-et-al-2010.git) containing the Codebook, the .csv file, the .html file, and the README.rmd file.

#### Accessibility

The data are openly available under an open-access license, Creative Commons Attribution 4.0 International. This allows unlimited access and use, if proper attribution has been given to the authors. The data can be downloaded using standard internet browsers without the need for special authentication. There should be minimal restrictions as all data is anonymized and does not contain personally identifiable information. The institutions that collected the original data are cited properly within the repository.

#### Interoperability

The data is stored in a generic .csv file to allow for compatibility across a variety of tools and software platforms. The variables were labeled using simple and descriptive names that inherently reflect the subject matter. No formal or structured ontologies were explicitly used; however terms were used to be consistent with common usage within this field of work. Additionally, the metadata is given in a README file and a codebook which allows clear interpretations and potential use of the data for further work.

#### Reusability

The data is under a clear open license (Creative Commons Attribution 4.0 International, CC BY 4.0), allowing others to reuse the current data. A detailed codebook has been attached in the repository as a pdf titled *Diener et al replication data codebook*. The codebook includes variable names, descriptions, value labels, data type, and category names for categorical variables. The dataset follows community standards related to the field, ensuring compatibility and ultimately reusability.

## (4) Reuse potential

These data could have several potential uses. First, researchers may explore relationships between variables using this dataset. With contribution from multiple international sites, the dataset offers a large sample with high variability, and generalizability across populations. It is also openly available to the public on our OSF page (https://osf.io/qdx7p/), enabling replication and secondary analyses. However, it is important to note that income data were not collected from all participants in several replications, which may impact certain analysis and aspects of data quality. Additionally, many of the participants were college-aged students regardless of data site, this would explain the lack of income data as many young adults might not have an income and participated in the studies.

These data can also be used for teaching purposes. They are well suited from use in introductory statistics courses to demonstrate correlation and regression analysis and may be valuable in social psychology classes exploring the relationship between types of needs and satisfaction.

## Footnotes

CREPCON is a free, virtual conference that began in 2022 to highlight the work of CREP students through talks and poster presentations.

## References

[1] Aknin, L. B., Dunn, E. W., Proulx, J., Lok, I., & Norton, M. I. (2020) Does spending money on others promote happiness? A registered replication report. Journal of Personality and Social Psychology, 119(2), e15–e26. 10.1037/pspa0000191 32250135

[2] Cantril, H. (1965). The pattern of human concerns. Cambridge University Press.

[3] Daniel, K., Alan, K. B., Norbert, S., & Arthur, S. A. (2004). A survey method for characterizing daily life experience: the day reconstruction method. Science, 306(5702), 1776–1780. 10.1126/science.1103572 15576620

[4] Diener, E., Ng, W., Harter, J., & Arora, R. (2010). Wealth and happiness across the world: material prosperity predicts life evaluation, whereas psychosocial prosperity predicts positive feeling. Journal of personality and social psychology, 99(1), 52–61. 10.1037/a0018066 20565185

[5] Disabato, D. J., Goodman, F. R., Kashdan, T. B., Short, J. L., & Jarden, A. (2016). Different types of well-being? A cross-cultural examination of hedonic and eudaimonic well-being. APA PsycNet, 28(5), 471–482. 10.1037/pas0000209 26348031

[6] Ford, Thomas E., Lappi, S. K., & Holden, C. J. (2016). Personality, humor styles and happiness: Happy people have positive humor styles. Europe’s journal of psychology, 12(3), 320. 10.5964/ejop.v12i3.1160 PMC4991042 27547251

[7] Grahe, J. E., McLaughlin, H., Psick, M., King, R., Haneda, R., Fung, H., … hesami, f. (2020, May 7). Replication of Diener, et al. (2010). at Pacific Lutheran University. Retrieved from https://osf.io/n3gyt

[8] Hatton, C. J., Grahe, J. E., & Gervais, H. (2019, February 5). Fork of Diener, E., Ng, W., Harter, J., & Arora, R. (2010). Retrieved from https://osf.io/q2th9

[9] Killingsworth, M. A., Kahneman, D., & Mellers, B. (2022). Income and emotional well-being: A conflict resolved. PNAS, 120(10), 1–6. 10.1073/pnas.2208661120 PMC10013834 36857342

[10] Legate, N., Johnson, K., Aguilar, A., Park, J., Chalasani, G., Ranginani, D., … Wisniewska, M. (2022, March 15). Wealth and Happiness: Replication of Diener, et al. at IIT 2019. Retrieved from https://osf.io/rhnyp

[11] Merkley, J., Kugan, M., Adamo, I., & yoon, h. (2019, April 5). CREP Diener Replication Project. Tutorial Section TUT5401. W19. Retrieved from https://osf.io/jc236

[12] Müller, M., Hartmann, A. J., Petersen, M., Ayrle, J., Panse, J., Pichler, M., … Filz, A. (2021, June 28). Wealth and Happiness: Replication of Diener, E., Ng, W., Harter, J., & Arora, R. (2010) at University of Vienna. Retrieved from https://osf.io/uzxyj

[13] O’Donnell, M., Dev, A. S., Antonoplis, S., Baum, S. M., Benedetti, A. H., Brown, N. D., Carrillo, B., Choi, A. L., Connor, P., Donnelly, K., Ellwood-Lowe, M. E., Foushee, R., Jansen, R., Jarvis, S. N., Lundell-Creagh, R., Ocampo, J. M., Okafor, G. N., Azad, Z. R., Rosenblum, M., … Nelson, L. D. (2021). Empirical audit and review and an assessment of evidentiary value in research on the psychological consequences of scarcity. Proceedings of the National Academy of Sciences, 118(44), e2103313118. 10.1073/pnas.2103313118 PMC8612349 34711679

[14] Pys, P. M., Young, B., Corzo, G., Legate, N., Shu, F., & Ranginani, D. (2021, June 25). Replication of Diener, Ng, Harter, & Arora (2010) at IIT Spring 2018. Retrieved from https://osf.io/knqe9

[15] Reed, Z., Haygood, P., Petit, T., Lang, B., & Egan, A. C. (2020, March 27). Replication of Diener et al. (2010) at Marian University. Retrieved from https://osf.io/w3qzg

[16] Srivastava, A., Locke, E. A., & Bartol, K. M. (2001). Money and subjective well-being: It’s not the money, it’s the motives. APA PsycNet, 80(6), 959–971. 10.1037/0022-3514.80.6.959 11414377

[17] Tian, Z., Metz, M., Qiu, Y., Yang, L., Li, Z., & Zhong, L. (2024, August 24). Replication of Diener et al. (2010) at University of Toronto, tut5401, W19. Retrieved from https://osf.io/bcz93

[18] Wagge, J. R., Brandt, M. J., Lazarevic, L. B., Legate, N., Christopherson, C., Wiggins, B., & Grahe, J. E. (2019). Publishing research with undergraduate students via replication work: the collaborative replications and education project. Front. Psychol, 247(10). 10.3389/fpsyg.2019.00247 PMC6381006 30814966

[19] Wong, C., & Law, W. (2019, December 31). (1) Materials. Retrieved from https://osf.io/yfj3d

