# Supplementary material for: Data from CREP Replication of Does Money Buy Happiness, Diener et al. (2010)
Source: J Open Psychol Data. 2026 Jun 15;14:2. doi: 10.5334/jopd.156 (PMC13281728; doi:10.5334/jopd.156)
Supplement: PR File 1. — Peer Review History. [file jopd-14-156-pr1.pdf]

Replication of Diener et al (2010) Reviewer Comments

Round 1

Editor Comments

Dear Olivia D Costa, Quincey V Feragen, Jordan Wagge, Amanda Mae Woodward,

After review, we have reached a decision regarding your submission to Journal of Open Psychology Data, " Data from CREP Replication of Does Money Buy Happiness, Diener et al. (2010)". Our decision is to request revisions of the manuscript prior to acceptance for publication.

The full review information is included at the bottom of this email. Please note that there is also a copy of the manuscript file with reviewer comments available once you have accessed the submission account. We ask you to please consider all the feedback and revise the file accordingly.

In summary, everyone is in agreement that this is high quality research and the dataset is valuable for reuse. It's a really exciting project and as a contributor to some CREP projects I am absolutely delighted you considered JOPD as a home for the dataset for this project. The reviewers raised some niggles with the presentation of the article and of the data files, and upon a further reading of the work with these comments in mind, I strongly believe revisions at this stage would be valuable and would make this work even stronger. I trust that these won't be too taxing but I do see great value in their feedback as they have been incredibly thorough. As such please can you follow the usual protocol with 'tracked changes' and a response to reviewer document as you consider whether, and if so how, to action each specific comment. Provided this goes as planned I will be happy to action the revisions myself without further review. If you have any questions in the meantime please don't hesitate to reach out.

Instructions for how to resubmit your article online are pasted below. Please ensure that your revised files adhere to our author guidelines, and that the files are fully proofed prior to upload. Please also include a revised version of your article with 'tracked changes', adding comments where appropriate, to indicate the revisions made, in addition to a brief document outlining how you have responded to the reviewers' requests.

If you have trouble processing the revisions, our Help Center (https://help.u-community.io) or downloadable PDF (https://bit.ly/Author-Guide-OJS-3) may be able to help. If not, please get in touch and we'll be happy to help.

Please also ensure that all copyright permissions have been attained for any figures/tables you have included.

Please could you have the revisions submitted with two weeks. If you cannot make this deadline, please let us know as early as possible.

Kind regards,

Prof Thomas Rhys Evans

You must upload your revised files and the response as follows:

1) login to the journal account with your username and password

2) access ‘My Queue’ and click ‘View’ for the submission in question

3) click ‘Upload File’, within the Revisions section

4) (a pop-up window will display) Select the article component you are uploading (e.g. manuscript, figure etc) and then either drag your file into the displayed area or click ‘Upload file’ to select it from your files. When done click ‘Continue’

5) check the file name is appropriate (edit it here if not) and then click ‘Continue’

6) if you wish to upload another revised file, click ‘Add another file’, otherwise, click ‘Complete’

If you need to upload more files, repeat steps 3-6.

If external review was obtained, the comments from the reviewers are below:

Reviewer 1:
Recommendation: Revisions Required

Comments to the author(s)

Summary
This paper describes a dataset created by combining data from all replications of Diener et

al (2010) through Project CREP. The dataset is relevant for researchers studying psychological well-being and for educational purposes.

Strengths

Despite the concerns raised below regarding framing and journal fit, the manuscript describes a dataset with several notable strengths. The data originate from a multi-lab replication project coordinated through CREP, enhancing transparency and cross-site consistency. The use of a well-established measurement framework based on Diener et al. (2010) increases the dataset's relevance and potential for reuse. In addition, the open availability of materials and data via OSF, along with the dataset's suitability for both research and teaching purposes, make it a potentially valuable resource for the community once the manuscript is revised to better align with the journal's focus on data description.

Points for Improvement

1. The manuscript is partly framed as a replication analysis rather than as a data descriptor. The OSF project description explicitly states, "Pooled analysis of the Diener et al. (2010) study. This analysis utilized the replication studies on OSF." If my understanding is correct, the primary aim of the JOPD is to document and disseminate information about existing datasets and their characteristics, rather than to report analytical or replication results. From this perspective, information related to replication outcomes should be omitted or substantially reduced. For example, Section 2.8 includes results of the replication analysis, and Table 2 reports analytical findings; both appear outside the scope of a data paper and could be removed. More generally, several sections are written from an analytical perspective (e.g., frequent references to "our analysis"). For instance: Section 2.2 ("Time and data collection") describes when the pooled analysis was conducted rather than only when the underlying data were collected. In Section 3.3 ("Data type"), the statement "Our analysis used secondary data found in OSF"does not fulfill the purpose of this section. Instead, this section should be strictly devoted to describing the technical and conceptual nature of the data (e.g., behavioral, self-report, physiological; participant-level vs. trial-level; variable types). Overall, the manuscript appears to have been originally written as an analysis report. I believe it could be made substantially more suitable for this journal by improving coherence and consistently aligning the text with the primary purpose of a JOPD data paper: the clear and neutral description of an existing dataset to facilitate reuse.

2. Abstract. The abstract is generally clear but is currently framed more like a study report than a data description. In particular, phrases such as "This study sought to understand..." emphasize research aims rather than the characteristics of the dataset itself. For a data paper in the JOPD, the abstract should focus on the provenance, structure, and type of data (e.g., self-report, participant-level, cross-sectional), as well as its potential for reuse, while avoiding references to hypotheses or analytical goals. Revising the abstract to more explicitly describe the dataset and its intended reuse would improve alignment with the journal's scope.

3. Background. The Background section is informative but currently reads partly like a study introduction and partly like a methods/results section. For a data paper in the JOPD, the background should primarily explain the context and rationale for the creation of the dataset, rather than summarize prior findings, describe analytical procedures, or report replication outcomes. In particular, reporting substantive findings is not necessary for motivating the dataset and may give the impression that the manuscript is framed as an analysis report rather than a dataset description. To better align with the journal's aims, the background could be streamlined to: briefly situate the dataset within the literature on income, needs, and well-being; explain why the Diener et al. (2010) framework is relevant; and clearly articulate the motivation for collecting and aggregating these replication datasets (e.g., educational value, and reuse potential). Refocusing the background on the reason for data collection and its anticipated reuse, rather than on analytical results or procedural detail, would substantially improve coherence and alignment with JOPD's scope.

4. In some places, the manuscript appears to reproduce the journal's template text verbatim rather than providing dataset-specific information. For example, Section 3.8 Publication date currently reads: "If already public, please state the date the dataset was published in the repository (11/12/2023)."

5. To ensure long-term findability and reuse, it may be advisable to host the dataset in a dedicated repository that is clearly distinguished from the pooled analysis project. The current OSF repository is titled "Pooled-Analysis of Diener: University of Minnesota–Twin Cities," which frames the content primarily as an analysis rather than as a standalone dataset (it includes a registration). Given that the aim of this manuscript is to describe an existing dataset for reuse, a separate repository (or at minimum a clearly labeled, dataset-focused component with its own README and metadata) would improve clarity, discoverability, and alignment with the journal's mission. This would also help future users distinguish between the raw/curated data and any analytical materials associated with the pooled analysis.

6. OSF repo. Similar to the point above, the repo includes results of statistical analyses, and a poster. This should be separated -- there should be a single repository describing only the data. The repo does not contain the README file. Neither json metadata. Files are duplicated, e.g., "replication_survey full export (1).pdf", the file names are not consistent (spaces, underscores). It also includes registration document. Further, the main file is proprietary excel instead of csv that is mentioned in the paper. It also includes data per site as a separate spreadsheets; for a properly formated dataset, it should be either a single document per dataset or a master document including all datasets but in a single spreadsheet. And what is this "Meta Analysis_ First Steps Spreadsheet.xlsx"? The codebook seems to be fine, but it may be easier to read if a table rather than a plain A4. Together, the repo appears unpolished.

7. In Section 3.9, the authors write: "The data is stored in a generic CSV file to allow for compatibility across a variety of tools and software platforms. The variables were labeled using simple and descriptive names that inherently reflect the subject matter. No formal or structured ontologies were explicitly used, however terms were used to be consistent with common usage within this field of work. Additionally, the metadata is given in a README

file and a codebook which allows clear interpretations and potential use of the data for further work." Maybe the link was given to the wrong repository?
8. Table 1. could provide more information about each dataset rather than just listing the sources. For example, number of participants, gender distribution and age, which measures are available, etc.
9. Survey instruments are described in a good way. Authors could include information about their reliability and validity. And perhaps potentially their correlations in a descriptive way. In general, I have the impression that the dataset (demographics, etc) is not sufficiently described. At the end of the day a reader should have a clear impression whether this is the dataset their can use for their particular purpose. This should be the primary objective of the authors when writing the manuscript.

Recommendation
Overall, this dataset has the potential to be a valuable resource, provided it is described and presented in a manner more closely aligned with the scope and aims of the journal.

Reviewer 2:
Recommendation: Revisions Required

Comments to the author(s)

Please see the attached file.

Round 2

Editor Comments

Dear Olivia D Costa, Quincey V Feragen, Jordan Wagge, Amanda Mae Woodward,

After review, we have reached a decision regarding your submission to Journal of Open Psychology Data, " Data from CREP Replication of Does Money Buy Happiness, Diener et al. (2010)". Our decision is to request very minor revisions of the manuscript prior to acceptance for publication.

The reviewers have provided some very minor little suggestions which might be worth considering - once you have actioned these as you see fit, please do resubmit and I will be happy to formally accept the manuscript for publication. Apologies this is a little cumbersome but it's easier than trying to make edits at a later stage. I don't need to see an author response document or such as the scope of the changes is so small. I hope this all helps and I can't wait to get this actioned through our production system!

If you have trouble processing the revisions, our Help Center (https://help.u-community.io) or downloadable PDF (https://bit.ly/Author-Guide-OJS-3) may be able to help. If not, please get in touch and we'll be happy to help.

Please also ensure that all copyright permissions have been attained for any figures/tables you have included.

Please could you have the revisions submitted with two weeks. If you cannot make this deadline, please let us know as early as possible.

Kind regards,

Prof Thomas Rhys Evans

You must upload your revised files and the response as follows:

1) login to the journal account with your username and password

2) access 'My Queue' and click 'View' for the submission in question

3) click 'Upload File', within the Revisions section

4) (a pop-up window will display) Select the article component you are uploading (e.g. manuscript, figure etc) and then either drag your file into the displayed area or click 'Upload file' to select it from your files. When done click 'Continue'

5) check the file name is appropriate (edit it here if not) and then click 'Continue'

6) if you wish to upload another revised file, click 'Add another file', otherwise, click 'Complete'

If you need to upload more files, repeat steps 3-6.

If external review was obtained, the comments from the reviewers are below:

Reviewer 1:
Recommendation: Accept Submission

Comments to the author(s)

I thank the authors for addressing all my points. Together with the suggestions of the other reviewer, the manuscript has substantially improved. Following these revisions, I believe the manuscript is suitable for publication.

I add only a few minor points. These are not criticisms, but rather suggestions for the authors to consider in finalizing the paper:
1. The abstract has improved compared to the previous version. However, given its importance for the paper's discoverability, I recommend reviewing it once more. For example, it contains several awkward or unclear phrases, particularly in the first sentence (e.g., "related to people's feelings and life evaluations in participants"), which affects readability. The writing is also somewhat redundant, with repeated references to "data/dataset" and some vague expressions (e.g., "a variety of ways").
2. I did not conduct a full reproducibility check. However, upon briefly and visually inspecting the CSV file, I noticed that some cells are empty. While I assume these indicate missing data, it may be preferable to mark them explicitly as NA values (provided this does not interfere with the interpretation of certain variables; in some cases, NA appears to be treated as a factor level). Additionally, the variable "household" appears to contain inconsistent formats: numeric values as well as strings such as "yes." In row 197 of this column, the income value seems to be repeated, and in row 418, the entry is "5 members." I am unsure whether these are intentional or typographical errors. More generally, it may be worth double-checking the accuracy and consistency of this file.
3. The file names referenced in Section 3.4 appear to differ from those available on OSF.

Reviewer 2:
Recommendation: Accept Submission

Comments to the author(s)

I read through the replies and skimmed the manuscript with tracked changes. All my points were adressed and resolved. I find additions to the manuscript are informative (e.g., Table 1) and think that the OSF repository is well-structured and easy to navigate. I found it a bit odd for the Readme to be an elaborate Rmd file instead of a concise list of information but think that it works in this case.
